# Atorvastatin pleiotropically decreases intraplaque angiogenesis and intraplaque haemorrhage by inhibiting ANGPT2 release and VE-Cadherin internalization

**DOI:** 10.1007/s10456-021-09767-9

**Published:** 2021-02-07

**Authors:** Fabiana Baganha, Rob C. M. de Jong, Erna A. Peters, Wietske Voorham, J. Wouter Jukema, Mirela Delibegovic, Margreet R. de Vries, Paul H. A. Quax

**Affiliations:** 1 Department of Vascular Surgery, Leiden University Medical Center, Leiden, The Netherlands; 2 Department of Vascular Surgery/Einthoven Laboratory for Experimental Vascular Medicine, Leiden University Medical Center, Leiden, PO Box 9600, 2300 RC Leiden, The Netherlands; 3 Department of Cardiology, Leiden University Medical Center, Leiden, The Netherlands; 4 Aberdeen Cardiovascular and Diabetes Centre, Institute of Medical Sciences, Aberdeen University, Aberdeen, UK

**Keywords:** Plaque angiogenesis, Plaque haemorrhage, Statins, Atherosclerosis, Vein graft disease, Statins

## Abstract

**Objective:**

Statins pleiotropically provide additional benefits in reducing atherosclerosis, but their effects on intraplaque angiogenesis (IPA) and hemorrhage (IPH) remain unclear. Therefore, we discriminated statin’s lipid-lowering dependent and independent effects on IPA and IPH.

**Approach and results:**

ApoE3*Leiden mice are statin-responsive due to ApoE and LDLR presence, but also allow to titrate plasma cholesterol levels by diet. Therefore, ApoE3*Leiden mice were fed a high-cholesterol-inducing-diet (HCD) with or without atorvastatin (A) or a moderate-cholesterol-inducing-diet (MCD). Mice underwent vein graft surgery to induce lesions with IPA and IPH. Cholesterol levels were significantly reduced in MCD (56%) and HCD + A (39%) compared to HCD with no significant differences between MCD and HCD + A. Both MCD and HCD + A have a similar reduction in vessel remodeling and inflammation comparing to HCD. IPA was significantly decreased by 30% in HCD + A compared to HCD or MCD. Atorvastatin treatment reduced the presence of immature vessels by 34% vs. HCD and by 25% vs. MCD, resulting in a significant reduction of IPH. Atorvastatin’s anti-angiogenic capacity was further illustrated by a dose-dependent reduction of ECs proliferation and migration. Cultured mouse aortic-segments lost sprouting capacity upon atorvastatin treatment and became 30% richer in VE-Cadherin expression and pericyte coverage. Moreover, Atorvastatin inhibited ANGPT2 release and decreased VE-Cadherin(Y685)-phosphorylation in ECs.

**Conclusions:**

Atorvastatin has beneficial effects on vessel remodeling due to its lipid-lowering capacity. Atorvastatin has strong pleiotropic effects on IPA by decreasing the number of neovessels and on IPH by increasing vessel maturation. Atorvastatin improves vessel maturation by inhibiting ANGPT2 release and phospho(Y658)-mediated VE-Cadherin internalization.

## Introduction

Statins are currently the principal drug in primary and secondary prevention of coronary artery disease [1]. As HMG-CoA-reductase inhibitors, they improve the human lipid profile and decrease atherosclerosis progression by lowering the low-density lipids (LDL) plasma levels [2, 3]. Interestingly, the observed benefits of statin treatment appear to be greater than what might be expected from changes in lipid levels alone, suggesting effects beyond cholesterol lowering. Indeed, many studies demonstrated that statins can pleiotropically improve endothelial cell (EC) function [4–7] and inflammation [8, 9], providing an additional benefit in the reduction of atherosclerosis [10–13].

Atherosclerotic plaques can grow to such dimensions that the core becomes hypoxic and intraplaque angiogenesis (IPA) is induced [14]. Angiogenesis, the physiological response to restore oxygen levels is a consistent feature of atherosclerotic plaque development [15]. Due to the growth of the intimal layer and the increased amount of metabolically active inflammatory cells in advance lesions, oxygen is consumed at a very high rate, triggering hypoxia inducible factor (HIF)1α activity and the expression of vascular endothelium growth factor (VEGF)A [16]. Consequently, ECs proliferate and migrate to form neovessel-like structures and overcome the oxygen demand in the plaque. However, these neovessels frequently have an immature nature, characterized by a discontinuous basement membrane, a lack of EC junctions and poor pericyte coverage [17]. Immature neovessels are highly susceptible to leakage of blood, resulting in intraplaque haemorrhage (IPH) [18]. Extravasated red blood cells are the main components of IPH and play a major role in cholesterol accumulation and monocyte recruitment into the plaque, initiating a vicious cycle that leads to further plaque destabilization [19].

Neovessel maturation is mainly regulated by the angiopoietin (ANGPT)1/2–Tie2 cascade [20, 21] ANGPT1 is an agonist of the Tie2 receptor and promotes the endothelial barrier function, by stimulating VE-Cadherin (VE-Cad) junctional accumulation in ECs and pericytes recruitment [22–26]. In hypoxia, ANGPT2 is rapidly released from EC’s Weibel–Palade bodies [27]. Excess of ANGPT2 antagonizes ANGPT1 [21], and inhibits Tie2 downstream signaling in pericytes and ECs, culminating in a phospho-dependent internalization of VE-Cad and pericyte recruitment inhibition [28]. Whereas ANGPT1 stabilizes vessels when angiogenesis is completed, ANGPT2 destabilizes the vasculature to potentiate VEGFA triggered angiogenesis [29].

In recent years, a growing number of studies has suggested that statins can exert anti-angiogenic effects and improve IPA [30–32]. It was shown that statins reduce adventitial neovascularisation in an ApoE^−/−^ mouse model [11], and IPA in the ApoE^−/−^Fbn1C1039G^+/−^ mouse model [12]. However, as observed in many other ApoE^−/−^ mouse models, the effects seen were not related to the cholesterol lowering capacity of statins since these strains are unable to respond to statins. In addition, in a clinical setting, it was reported that patients treated with atorvastatin present less IPA compared to non-treated patients [33]. However, for those reported effects, it could not be discriminated whether they were lipid-lowering dependent or independent. Therefore, the extent of a potent benefit of the pleiotropic effects of statin therapy in IPA and IPH still remains to be determined and especially the molecular mechanism(s) behind it.

Mouse models commonly used to study atherosclerosis are deficient in LDLR or ApoE, both crucial proteins in LDL clearance. Their absence makes it impossible for statins to rescue LDL from the blood, since the inhibition of HMG-CoA reductase (the mode of action of statins) does not occur. As a result of the inhibition of the intracellular synthesis also the uptake of LDL in the blood fails in ApoE^−/−^ and LDLR^−/−^ mice. Thus, no changes in blood lipid levels are found in ApoE^−/−^ and LDLR^−/−^mice upon statin treatment. Additionally, spontaneous murine atherosclerotic lesions are hardly suitable to study IPA. Due to their small size lesions, no ischemia occurs, resulting in none to minimal neovessels infiltration. To overcome these issues, we used hypercholesterolemic ApoE*Leiden mice and the vein graft model to study the pleiotropic effects of statins on IPA and IPH. ApoE3*Leiden mice are a transgenic strain that contains the human ApoE3*Leiden gene and develops hyperlipidaemia due to the defective binding of ApoE3*Leiden to the LDLR [34, 35]. This mutation makes it possible to create a diet-induced human like atherosclerotic plaque while these mice keep the sensitivity to statins due to the presence of ApoE and LDLR. Moreover, as we previously reported, the vein graft model presents plaque components that highly resembles human plaque features, including ischemia, plaque angiogenesis and leaky neovessels [36].

In this study, we demonstrate both, the lipid lowering-dependent and independent effects of atorvastatin on vein graft atherosclerosis, including IPA and IPH. We also present evidence of the pathophysiological and molecular mechanism of atorvastatin-mediated inhibition on neovascularization.

## Material and methods

### Animals

All animal experiments were performed in compliance with the Animal Welfare Committee of the Leiden Medical University Center (project number: 13064) and the Directive 2010/63/EU of the European Parliament. Male ApoE3*Leiden mice, crossbred in our own colony on a C57BL/6 background for at least 40 generations, 10–16 weeks old, were allocated randomly to three groups. One group was fed with a moderate-cholesterol inducing diet (Diet W: 1% cholesterol and 0.1% cholate w/w, AB diets)—MCD group—and the other two with a high-cholesterol inducing diet (Diet N: containing 1% cholesterol and 0.1% cholate w/w, AB diets) with and without atorvastatin (2.8 mg/kg/day)—HCD + A and HCD groups, respectively—during all the experiment. Food and water were provided ad libitum and the mice were housed on regular bedding and nesting material.

### Vein graft surgery

After three weeks on respective diets, the mice underwent vein graft surgery, in which a donor caval vein, derived from a non-transgenic litter, was placed as an interposition in the carotid artery of recipient mice, as described before [36]. Mice were anesthetized via intraperitoneal injection of 5 mg/kg midazolam (Roche Diagnostics), 0.5 mg/kg medetomidine (Orion Corporation) and 0.05 mg/kg fentanyl (Janssen Pharmaceutical). After the surgery, the anaesthesia was antagonized with 2.5 mg/kg atipamezol (Orion Corporation) and 0.5 mg/kg fluminasenil (0.5 mg/kg, Fresenius Kabi). 0.1 mg/kg buprenorphine (MSD Animal Health, Netherlands) was given for pain relieve. On the day of sacrifice mice underwent deep anesthesia and were euthanized by exsanguination, followed by 3 min of in vivo perfusion-fixation with PBS and 4% formaldehyde (100,496, Sigma-Aldrich). The vein grafts were harvested and fixed in 4% formaldehyde. Blood samples were collected and plasma cholesterol levels of all mice were determined before and 28 days after the vein graft surgery (1,489,437, Roche Diagnostics).

### Histological analysis of the vein grafts

Vein graft samples were embedded in paraffin and sequential cross Sections (5 µm thick) were taken from the entire length of the cuffed vein. For each mouse, six equal spaced cross-sections over the total vein graft length were used for analysis.

To assess vein graft remodeling morphometry was performed on the cross sections. For this, the vein graft area surrounded by the adventitia (*Vessel Area*), the area of the lumen (*Lumen Area*) and *Vessel Wall Area* (subtraction of the last two) were measured in Hematoxilin–Phloxin–Saphron stained sections.

To assess vein graft morphology, the amount of collagen (% *Collagen*), by staining for Sirius Red, and the presence of vascular smooth muscle cells (% *VSMCs*) and macrophages (% *Macrophages*), by immunohistochemistry for alpha smooth muscle actin (αSMA, 1A4 Dako) and Mac-3 (553,322, BD Pharmingen), were quantified.

To assess intraplaque angiogenesis and intraplaque hemorrhage, a combined immunofluorescence staining was performed for CD31 (sc-1506-r, Santa Cruz, Biotechnology), to detect neovessels, for αSMA, to evaluate vessel maturation, and for TER119 (116,202, Biolegend) to rate endothelium leakage. CD31 + neovessels structures were manually counted (% Neovessels) and the percentage of neovessel CD31 + αSMA- was defined as % Immature Neovessels. Intraplaque Hemorrhage was regionally assessed using a scoring system accounting for the presence and the number of erythrocytes outside the neovessels. No presence was scored as 0, low number of erythrocytes outside the neovessels (1–10) was score as 1, intermediate number (11–30) as 2, high number (> 30) as 3.

For each antibody, an isotype-matched was used as a negative control. The measured immuno-positive area is expressed as a percentage of the Vessel Wall Area. Pictures were acquired with the Pannoramic SCAN II (3DHistech) and analyzed with QWIN software (Leica).

### Cell culture

For the isolation of HUVECS anonymous umbilical cords were obtained in accordance with guidelines set out by the ‘Code for Proper Secondary Use of Human Tissue’ of the Dutch Federation of Biomedical Scientific Societies (Federa), and conform to the principles outlined in the Declaration of Helsinki. Human umbilical vein endothelial cells (HUVEC) were isolated and cultured as described by Welten et al. [37]. In brief, the vein in the umbilical cords was flushed with PBS and incubated with 0.75 mg/mL collagenase type II (LS004177, Worthington Biochemical Corporation) for 20 min at 37 °C. Detached ECs were washed out of the vessel and left to grow in complete medium [EBM-2 medium (00,190,860) supplemented with EGM BulletKit (CC-3124, Lonza) and 2% of FBS (10,082,139, ThermoFisher Scientific)] at 37 °C in a 5% CO_2_ humidified incubator. Culture medium was refreshed every 2–3 days. Cells were passed using trypsin–EDTA (T4049, Sigma-Aldrich) at 90–100% confluency. HUVECs were used up to passage three for proliferation and migration assays, and up to passage seven for protein expression analysis by western blotting.

### Metabolic assay

Cell metabolism was measured by the reduction of (3-(4,5-dimethylthiazol-2-yl)-2,5-diphenyltetrazolium bromide (MTT, M5655, Sigma-Aldrich). HUVECs were seeded in 96-wells plate in complete medium and grown until 80% confluency. To cause cell cycle arrest, cells were incubated for 24 h in EBM-2 medium supplemented with 0.2% FBS. Atorvastatin was added in a concentration range of 0.05 µg/ml, 0.5 µg/ml until 5 µg/ml. After 18 h, cells were incubated with MTT for 4 h. A supernatant fraction was replaced by 0.01 N HCL-isopropanol (258,148 and 563,935, Sigma-Aldrich) and absorbance was measured at 570 nm by Cytation™ 5 Cell Imaging Multi-Mode Reader (BioTek Instruments).

### Migration assay

For migration assays, HUVECs were seeded in 12-wells plate in complete medium and grown until 80% confluence. To cause cell cycle arrest, cells were incubated in EBM-2 medium supplemented with 0.2% FBS and 24 h later, a scratch-wound was made. Atorvastatin was added in a concentration range of 0.05 µg/ml, 0.5 µg/ml until 5 µg/ml. Three locations along the scratch-wound were marked per well and scratch-wound closure at these sites was imaged at time 0 and 18 h by using an Axiovert 40c Inverted & Phase Contrast Microscope (451,207, Carl Zeiss). Average scratch-wound closure was calculated by measuring cell coverage at 18 h vs 0 h using ImageJ.

### Aortic ring sprouting assay

The aortic ring assay was performed as described previously.[37–39] Three ApoE3*Leiden mice, 4–8 weeks old, were anesthetized and the aorta was dissected. Each aorta was cut in 1 mm rings, and serum-starved in Gibco™ Opti-MEM™ GlutaMAX (51,985,034, ThermoFisher Scientific) overnight at 37 °C and 5% CO2. On the next day, each ring was mounted in a well of a 96-well plate in 70 µl of 1.0 mg/ml acid-solubilized collagen type-I (11,179,179,001, Roche Diagnostics) in DMEM (12,634,010, ThermoFisher Scientific). After collagen polymerization, Gibco™ Opti-MEM™ GlutaMAX supplemented with 2.5% FCS and 30 ng/ml VEGF (293-VE, R&D systems) was added with atorvastatin (0.05 µg/ml, 0.5 µg/ml and 5 µg/ml). The rings were cultured for 7 days and photographed by using an Axiovert 40c microscope. The number of sprouts were counted manually. For immunohistochemistry, rings were formalin-fixed and permeabilized with 0.2% Triton X-100 (11,332,481,001, Merck). Rings were stained with αSMA, CD31 and VE-Cadherin (AF1002, R&D Systems). Extended focus pictures were made with the Pannoramic SCAN II and quantified with Image J.

### Protein expression analysis by western blotting

HUVECs (80% confluent) were treated overnight with increasing doses of atorvastatin (0.05 µg/ml, 0.5 µg/ml and 5 µg/ml) in EBM-2 medium with 0.2% FBS and stimulated for 30 min with 50 ng/ml of PMA.

Medium was collected for TCA-precipitation [40] and cells were scraped in RIPA buffer [10 mM Tris–HCl pH = 7.4, 150 mM NaCl, 5 mM EDTA pH = 8.0, 1% Triton X-100, 1% SDS, with freshly added 1 mM NaF, 1 mM Na3VO4 and cOmplete™ Protease Inhibitor Cocktail (1,169,749,800, Roche Diagnostics)]. Total protein concentration was quantified by Pierce™ BCA Protein Assay Kit (23,225, ThermoFisher Scientific). 15 µg of protein were separated by SDS-PAGE in a 4–15% minigel and transferred into a nitrocellulose membrane. Blots were incubated with pY685-VE-Cadherin (ab119785, Abcam); VE-Cadherin (MA1-198, ThermoFisher), pY992-Tie2 ((4221S, Cell Signaling Technology); Tie2 (4224S, Cell Signaling Technology); pY418-Src (ab4816, Abcam); Src (2110, Cell Signaling Technology); ANGPT2 (ab155106, Abcam)) overnight at 4ºC. A suitable peroxidase conjugated secondary antibody was used (31,462, 31,432, 31,400, ThermoFisher Scientific). Proteins of interest were imaged with SuperSignal™ West Pico PLUS Chemiluminescent Substrate (34,580, ThermoFisher Scientific) and the ChemiDoc™ Touch Imaging using System (1,708,370, Bio-Rad Laboratories). β-actin (ab8220, Abcam) was used as internal control and blots were quantified with Image J.

### Statistical analysis

All data are presented as mean ± standard error of the mean (SEM). Normality was determined using the Shapiro–Wilk normality test. Overall comparisons between groups were performed using 1-way ANOVA on parametric data using the statistics software GraphPad Prism 8.02. *p* values less than 0.05 were regarded as statistically significant.

## Results

### Atorvastatin decreases cholesterol levels in ApoE3*Leiden mice

**Fig. 1:**
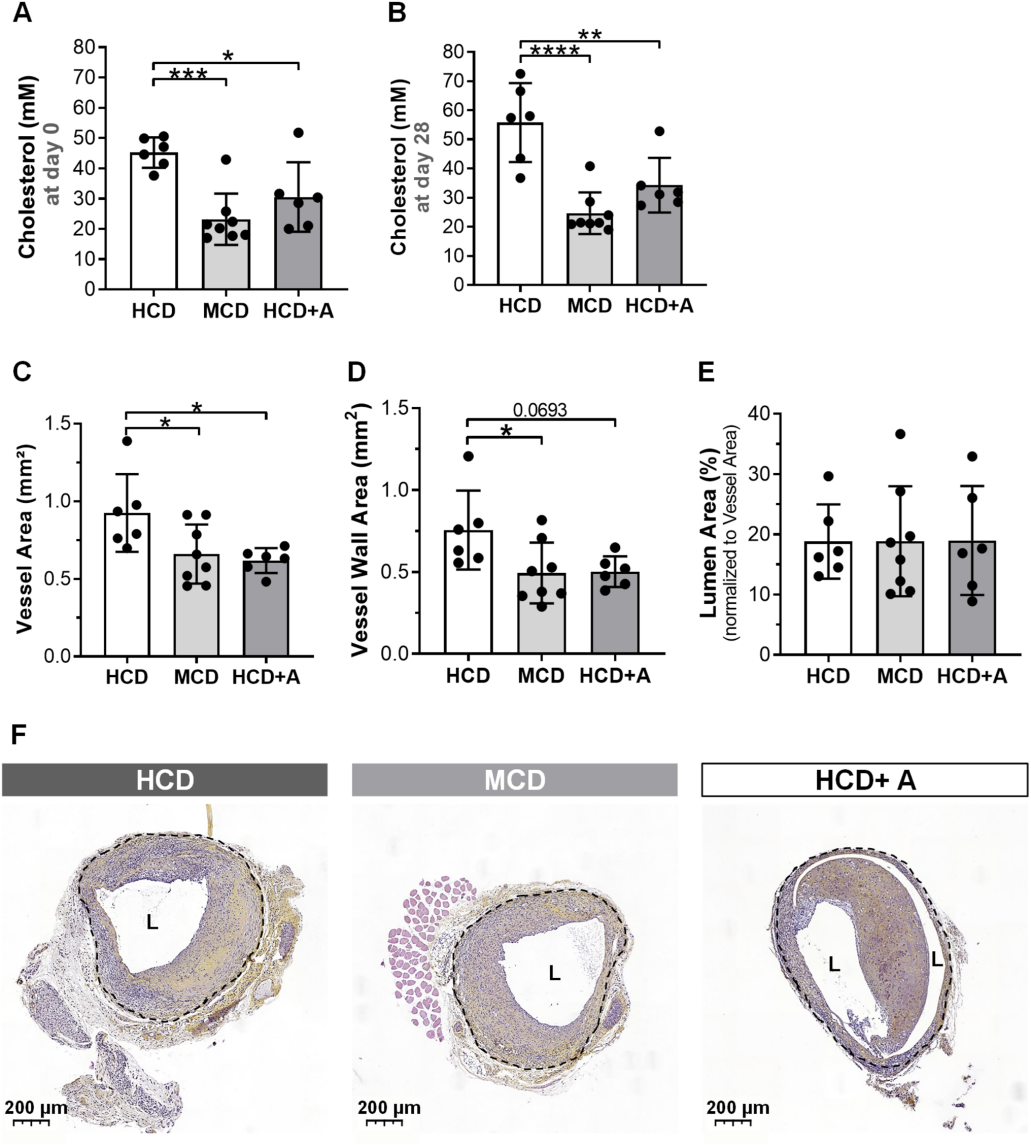
Atorvastatin reduces cholesterol levels and vein graft thickening in ApoE3*Leiden mice. Cholesterol levels one day before surgery (**a**) and at day of sacrifice (**b**). Quantitative measurements of Vessel Area (**c**), Vessel Wall Area (**d**) and Lumen Area (**e**). Representative vein grafts cross sections of an Haematoxylin–Phloxine–Saffron staining in HCD (*n* = 6), MCD (n = 8) and HCD + A (*n* = 6) (**f**). Data presented as mean ± SEM. *p ≤ 0.05, **p ≤ 0.01, ***p ≤ 0.001, ****p ≤ 0.0001 by 1-way ANOVA

To evaluate the cholesterol levels in ApoE3*Leiden mice on a moderate-cholesterol inducing diet (MCD) and on a high-cholesterol inducing diet (HCD) with or without atorvastatin (A), we measured the plasma cholesterol levels one day before vein graft surgery (Fig. 1a) and 28 days after surgery, at terminal day (Fig. 1b).

At both time points, we observed a comparable degree of cholesterol lowering. At day 1, the plasma cholesterol levels were in the MCD (by 49%, *p* = 0.0006) and in the HCD + A (by 32%, *p* = 0.0190) groups in comparison to the HCD group. At day 28, those plasma cholesterol levels were further decreased in the MCD (by 56%, *p* < 0.0001) and HCD + A (by 39%, *p* = 0.0035) groups, respectively, in comparison to the HCD group. At both time points, no significant differences were observed between the MCD and HCD + A groups.

Therefore, these ApoE3*Leiden mice can be used to study the pleiotropic effects of atorvastatin on vein graft lesions, an accelerated form of atherosclerosis, by comparing the effects of atorvastatin in the HCD + A group with the effects of lipid lowering alone, as is observed in the MCD group.

### Atorvastatin decreases vein graft thickening

To assess vein graft lesion morphometry, we measured Vessel Area, Vessel Wall Area and Lumen Area as represented in Fig. 1.

Both, MCD and HCD + A groups, show a decrease in *Vessel Area* in comparison to HCD group, by 29% (*p* = 0.0361) and 33% (*p* = 0.0346), respectively (Fig. 1c and f). Atorvastatin treatment resulted in a comparable *Vessel Area* to the MCD group. Both MCD and HCD + A groups revealed a decrease in *Vessel Wall Area* in comparison with HCD group, 35% (*p* = 0.0430) and 34% (*p* = 0.0693), respectively (Fig. 1d and f). Whilst, no significant differences between MCD and HCD + A groups were observed. The *Lumen Area* was similar in all groups (Fig. 1e and f).

Since no differences were observed between HCD + A and MCD groups, and both exhibited similar cholesterol profiles, the observed effects on vein graft remodeling, as detected by morphometry, were most likely due to the lipid-lowering effects of atorvastatin.

### Atorvastatin decreases inflammation

**Fig. 2:**
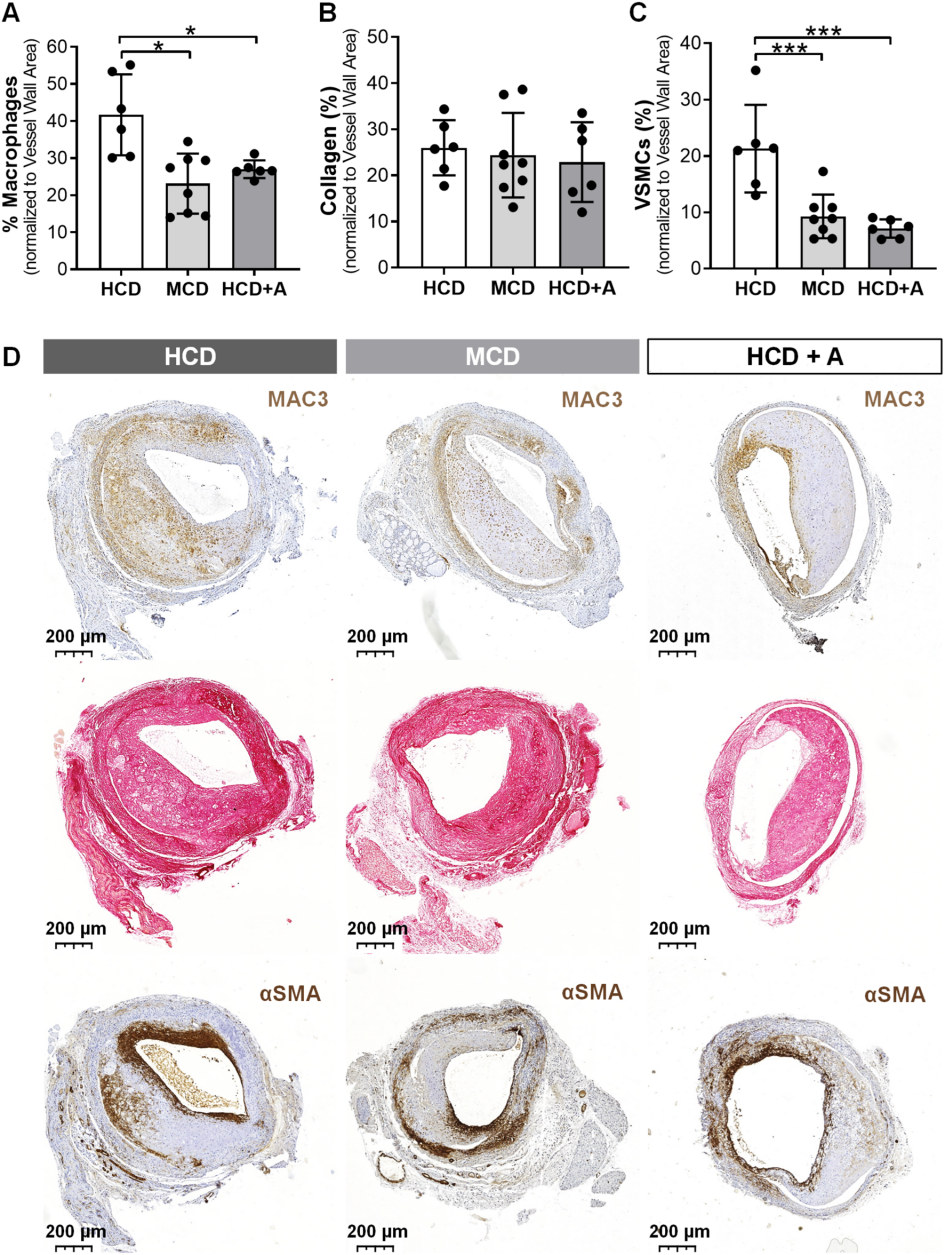
Atorvastatin increases plaque stability in ApoE3*Leiden mice. Quantitative measurements of % *Macrophages* (**a**), *% Collagen* (**b**) and *% VSMCs* (**c**). Representative vein grafts cross sections of Mac3, Sirus Red and αSMA stainings of HCD (*n* = 6), MCD (*n* = 8) and HCD + A (*n* = 6) group (**d**). Data presented as mean ± SEM. *p ≤ 0.05, ***p ≤ 0.001 by 1-way ANOVA

To assess plaque morphology, we evaluated lesion inflammation by determining the percentage (%) Macrophages and lesion stability by determining the % VSMCs and % Collagen in the vessel wall (Fig. 2).

The % Macrophages was lower in the MCD (by 45%, *p* = 0.0013) and in the HCD + A (by 36%, *p* = 0.0142) groups in comparison to the HCD group (Fig. 1a and d). Atorvastatin-treated group had a comparable % Macrophages as the MCD group. The % Collagen did not vary between the groups (Fig. 2b and d). Both MCD and HFD + A groups exhibited a highly significant decrease in the % VSMCs compared with the HCD group, 56% (*p* = 0.0007) and 67% (*p* = 0.0004), respectively, but the MCD and HCD + A groups had similar % VSMCs values (Fig. 2c and d).

Since % Macrophages and % VSMCs were equal in the HCD + A and MCD, and both groups had comparable cholesterol profiles, this decrease in macrophages and VSMCs in vein graft lesions could again be attributed to the lipid-lowering effect of atorvastatin.

### Atorvastatin decreases plaque neovessel density, increases vessel maturity, improving intraplaque hemorrhage in vivo

**Fig. 3:**
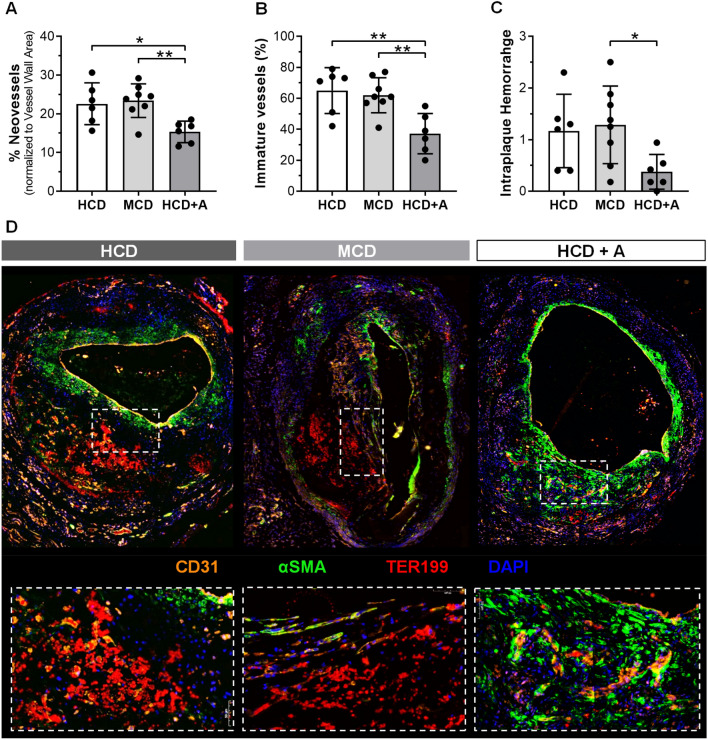
Atorvastatin reduces plaque neovessels density and vessel immaturity, decreasing intraplaque haemorrhage in vivo*.* Quantitative measurements of % *CD31*^+^ *Neovessels* (**a**) and % *Immature Neovessels* (**b**) and Intraplaque Haemorrhage scoring (**c**). Representative vein grafts cross sections of CD31 (orange), αSMA (green), TER119 (red) and DAPI (blue) staining in HCD (*n* = 6), MCD (*n* = 8) and HCD + A (*n* = 6) groups (**d**). Data presented as mean ± SEM. **p ≤ 0.01, ***p ≤ 0.001, ****p ≤ 0.0001 by 1-way ANOVA

To evaluate intraplaque angiogenesis and intraplaque hemorrhage, we measured % *Neovessels*, % *Immature Neovessels* and scored *Intraplaque Hemorrhage* in the vein graft lesions (Fig. 3).

The *% Neovessels* was decreased by 30% in atorvastatin-treated group, when compared with the HCD (*p* = 0.0472) and MCD (*p* = 0.0395) groups, as shown in Fig. 3a. The HCD and MCD groups presented similar *% Neovessels* values. For studying (im)maturity of the neovessels, we determined the coverage of the neovessels by pericytes. In Fig. 3f, we revealed that neovessels (CD31, in orange) from plaques treated with atorvastatin were surrounded by a more continuous pericyte coverage (αSMA, in green) when compared with the other groups.

Quantification of *% Immature vessels* (Fig. 3c) showed a decrease in atorvastatin-treated group, when compared with the HCD (by 34%, *p* = 0.0002) and MCD (by 25%, *p* = 0.0066), with no differences between the last two groups.

We also observed that neovessels that were partly devoid of pericyte coverage were more prone to leakage of the erythrocytes (Ter199, in red) in Fig. 3d. Accordingly, *Intraplaque Hemorrhage* was less present and less severe in the atorvastatin-treated group when compared with the HCD and MCD (*p* = 0.0455) groups (Fig. 3d).

Taken together, our findings suggest that atorvastatin reduces intraplaque angiogenesis and intraplaque hemorrhage independent of its lipid lowering effects in vivo.

### Atorvastatin decreases EC proliferation and migration in vitro

To confirm the pleiotropic effect of atorvastatin on intraplaque angiogenesis, we studied the effects of increasing doses of atorvastatin (0.05 µg/ml, 0.5 µg/ml and 5 µg/ml) on the capacity of HUVECs to proliferate and migrate.

**Fig. 4:**
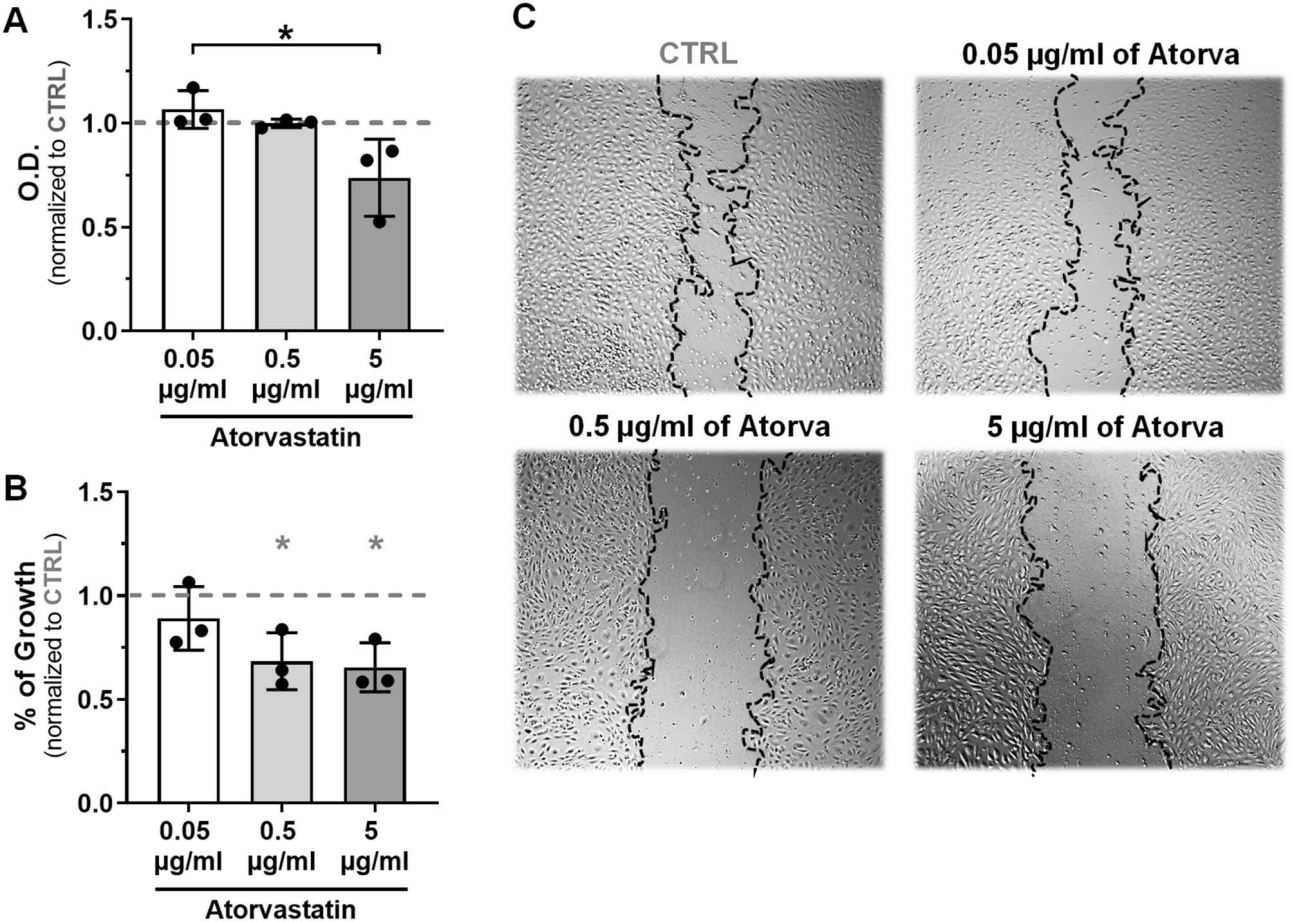
Atorvastatin reduces EC proliferation and migration in vitro*.* Quantification of Atorvastatin effects on MMT assay (**a**) and on scratch wound healing assay (**b**) and representative images of wounds treated with increasing doses of Atorvastatin and control (**c**), 18 h after scratching. Data normalized to CTRL group (indicated as 1 by a dashed grey line in the graphs) and presented as mean ± SEM (*n* = 3). *p < 0.05, **p < 0.01; by 1-way ANOVA (* (in grey) are significances vs CTRL; * (in black) are significances between groups)

ECs proliferation (Fig. 4a) was significantly decreased by 26% in 5 µg/ml dose of atorvastatin (*p* = 0.0197).

Regarding ECs migration (Fig. 4b and c), the ability of wound closure was dose-dependently decreased by atorvastatin when compared to control, with significant effects at 0.5 µg/ml (32% reduction, *p* = 0.0453) and 5 µg/ml (35% reduction, *p* = 0.0299).

These results suggest that atorvastatin decreases EC pro-angiogenic behavior in vitro.

### Atorvastatin decreases sprouting while increases VE-Cad expression and pericyte coverage

**Fig. 5:**
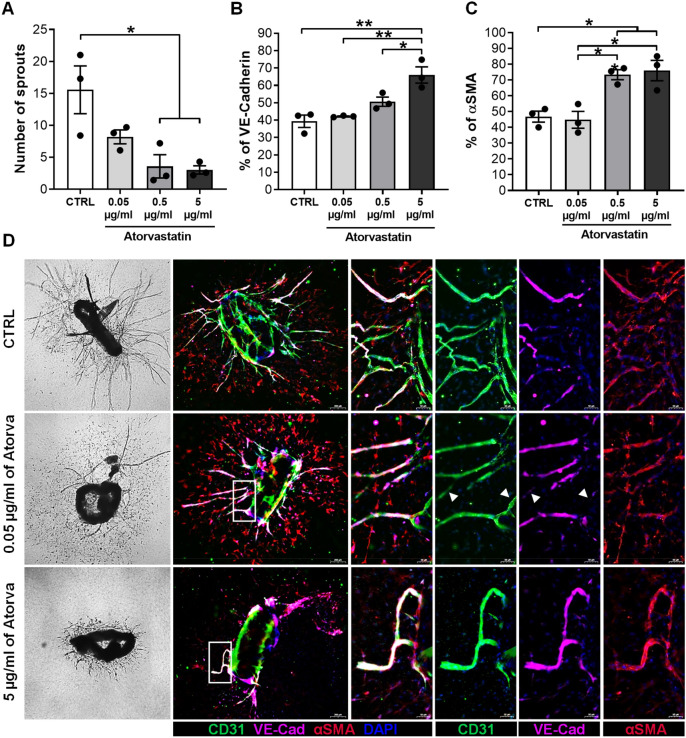
Atorvastatin reduces sprout formation while improves vessel maturation by increasing VE-Cadherin expression ex vivo*.* Quantification of the *Number of Sprouts* after treatment with increasing doses of Atorvastatin and VEFG (30 ng/ml) and control group treated only with VEGFA (30 ng/ml) in an ex vivo aortic ring assay (**a**). Phase-contrast representative images of aortic rings showing microvessel outgrowth (**d**). Data is presented as mean ± SEM of 15 aortic segments per treatment, which were cut from aortas of three different mice. Quantification of % *VE-Cad* (**b**) and % *αSMA* (**c**) along the sprout. Representative examples (**d**) of extended focus scanner images of the aortic ring sprouts fluorescently stained for: **CD31** (green) stains endothelial cells; **VE-Cad** (pink); **α-SMA** (red) stains smooth muscle cells and **DAPI** (blue). Data presented as mean ± SEM (*n* = 3). *p < 0.05, **p < 0.01; by 1-way ANOVA

To study the pleiotropic effects of atorvastatin on neovessel sprouting, we cultured mouse aortic segments ex vivo with increasing doses of atorvastatin: 0.05 µg/ml, 0.5 µg/ml and 5 µg/ml (Fig. 5). The number of sprouts formed was decreased dose-dependently by atorvastatin when compared to the control group, with a 5-fold decrease for 5 µg/ml (*p* = 0.0150) treated segments (Fig. 5a, d, f, h).

Next, we performed, on the same aortic segments, a triple staining for CD31, αSMA and VE-Cad to assess vessel maturation (Fig. 5e, g, f). In concordance with the previous quantification (Fig. 5a), we observed less CD31 + sprouts in the treated groups compared to the control group (Fig. 5e, g, i). Additionally, we determined that sprouts has increased VE-Cad levels when treated with atorvastatin (Fig. 5E1-3, G1-3, I1-3).

Quantification of VE-Cad expression revealed a dose-dependently increase in expression by atorvastatin, in comparison to the control group, with significant effects at 5 µg/ml (27%, *p* = 0.0017). In parallel, as the concentration of atorvastatin increased, αSMA + cells along the sprouts were more abundant and organized (Fig. 5E4, G4, I4).

Quantification of our data revealed that αSMA^+^ cell presence significantly increased when treated with 0.5 µg/ml (by 26%, p = 0.0187) and 5 µg/ml (by 29%, *p* = 0.0112) of atorvastatin, compared to the control group (Fig. 5c). In summary, atorvastatin not only decreased vessel sprouting, but also increased vessel maturation ex vivo.

### Atorvastatin increases vessel maturation, by inhibiting ANGPT2 release and decreasing VE-Cad(Y658)-phosphorylation

To elucidate the molecular mechanism(s) on vessel maturation, we investigated the effects of atorvastatin on the ANGTP1/2-TIE2 signaling pathway.

**Fig. 6:**
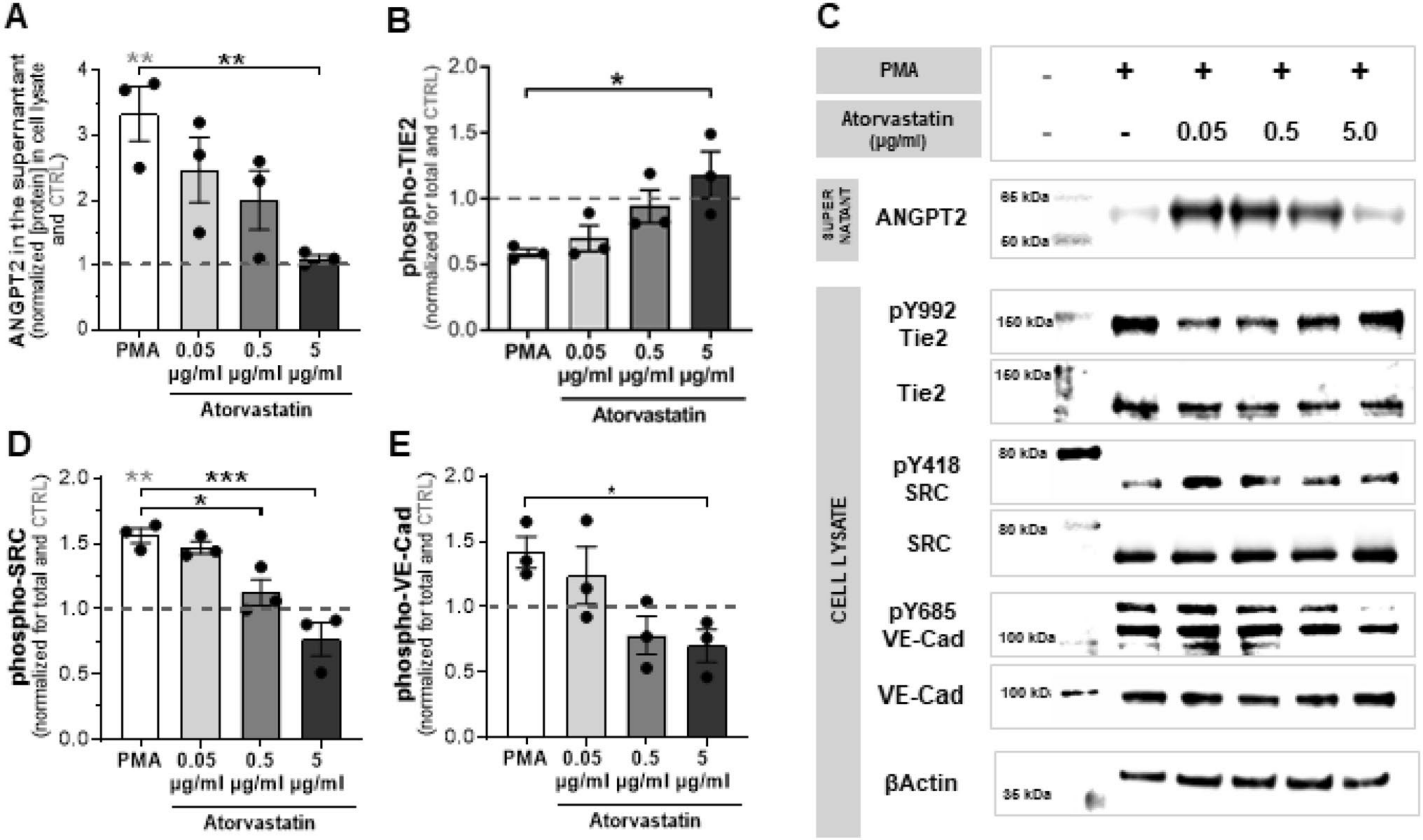
Atorvastatin increases vessel maturation by inhibiting ANGPT2 release. Quantification of ANGPT2 levels in medium (**a**) of HUVECs monolayers, treated overnight with Atorvastatin (0.05, 0.5 or 5 µg/ml) and stimulated with PMA (50 ng/ml) for 30 min. Quantification of phospho TIE2 (**b**) VE-Cad (**d**) an Src (**e**) relative to their total levels in the cell lysates. Representative experiment is shown on **C**. Expression was normalized to the CTRL (indicated as 1 by a dashed grey line) and presented as mean ± SEM (*n* = 3). *p < 0.05, **p < 0.01; ***p < 0.001, 1-way ANOVA *(in grey) are significances vs CTRL; * (in black) are significances between groups

Unstimulated cells released low levels of ANGPT2, however, upon stimulation with PMA [27, 41, 42], ANGPT2 levels in the medium increased significantly (Fig. 6a and c). Such release was lowered, in a dose–response manner, reaching basal levels at 5 µg/ml of atorvastatin (*p* = 0.009). When studying the activation of the Tie2 receptor by quantifying its Y992 phosphorylation, we observed that 5 µg/ml atorvastatin dose completely restored phosphorylation of the receptor. In fact, ANGPT2 and Y992-Tie2 levels were inversely correlated, suggesting that atorvastatin restores Tie2 phosphorylation by preventing ANGPT2 release.

Next, we assessed VE-Cad endocytosis by quantifying the phosphorylation of Y418 of Src and Y685 of VE-Cad. As predicted, inhibition of the Tie2 receptor (by ANGPT2 release) induced Src and VE-Cad phosphorylation (Fig. 6d, e, c). These phosphorylations were decreased in a dose-dependent manner, by atorvastatin, reaching basal levels at 5 µg/ml, for both proteins (Src-Y418: *p* = 0.0003; VE-Cad-Y685: *p* = 0.0317). These findings suggest that atorvastatin is able to prevent VE-Cad internalization by decreasing its Src-dependent phosphorylation.

Taken together, our results suggest a mechanism by which atorvastatin increases vessel maturation, by inhibiting ANGPT2 release, a vascular-destabilizing factor, and decreasing VE-Cad Y658-phosphorylation, a key EC junction.

## Discussion

In the current study, we demonstrated that atorvastatin has a beneficial effect on atherosclerotic plaque stability in hypercholesterolemic ApoE3*Leiden vein grafts due to its lipid lowering capacity. Furthermore, we demonstrated that atorvastatin has strong pleiotropic effects not only on IPA by reducing the number of intraplaque neovessels but also on IPH by increasing vessel maturation. As a mechanism, we provided evidences that atorvastatin affects neovessel stabilization by increasing pericyte coverage and EC junctions presence. We demonstrated that atorvastatin inhibits ANGPT2 release (a vascular-destabilizing factor), restoring Tie2-receptor activation (the main receptor in vessel maturation) and decreasing VE-Cad Y658-phosphorylation (a key EC junction).

Remodeling of the vessel wall is a crucial process in the development of atherosclerotic lesions in vein grafts [43]. During the adaptation of the venous segment to the arterial blood pressure, VSMCs from the media proliferate and migrate leading to the growth of the intimal layer. In this study, we observed that atorvastatin treatment reduces outward remodeling or vessel area and lesion size or vessel wall area when compared to the HCD group, while no differences in lumen area were found between the groups. Similar effects were observed between the MCD and HCD group. Because HCD + A and MCD groups exhibited equal cholesterol levels, the decrease in outward remodeling and lesion size could thus be attributed to atorvastatin mediated cholesterol lowering dependent effects. This is in concordance with other studies that demonstrated that statins decrease intimal hyperplasia [11, 44, 45]. Changes in the plaque composition such as a reduction in the percentage of macrophages and VSMCs were observed to the same extend in the HCD + A and MCD group, when compared to the HCD. Since these effects were observed in both groups, these effects are cholesterol dependent. Macrophages are important in vein graft remodeling and vein graft plaque stability especially via their secretion of chemokines [43]. Therefore, decrease in macrophage content, which has been also reported by others as a statin-mediated effect [12, 46], contributes to plaque stability.

In this study, we demonstrate that atorvastatin decreases IPA in ApoE3*Leiden mice. This finding is documented in a set-up where ApoE3*Leiden mice cholesterol levels were not only significantly lowered upon atorvastatin treatment, but at a similar level as the MCD control group. Therefore, we were able to study statins’ cholesterol-independent effects in a clinically relevant way. Vein graft lesions treated with atorvastatin showed significantly less CD31^+^ neovessels not only when compared with the HCD group but, more importantly, also compared to the MCD group. Furthermore, we showed that atorvastatin decreased proliferation and migration of human vascular endothelial cells in vitro and decreases capillary formation in an ex vivo aortic ring sprouting assay. These findings are in line with other reports in which statins have been shown to have anti-angiogenic effects. For instance, a reduction in cell proliferation and migration has been associated with a decrease in the production of essential signaling proteins in cytoskeleton function when cholesterol synthesis was inhibited [47]. Moreover, it has been reported that in patients with coronary artery disease, atorvastatin treatment lowered VEGF expression [48] which was also observed in diabetic and non-diabetic rats [49]. In addition, tumors treated with simvastatin showed lower HIF‐1α and VEGF levels [50].

Another novel aspect of our results relates to the effects of atorvastatin on vessel maturation. We and others have previously shown that neovessels in advanced atherosclerotic lesions are structurally vulnerable due to a lack of perycytes [17, 51, 52]. Here, we demonstrated that plaque neovessels in the HCD and MCD groups are characterized by poor pericyte coverage, in contrast to neovessels in HCD + A group which are rich in enveloping pericytes. Subsequent quantification demonstrated that atorvastatin significantly decreased the % Immature Neovessels in comparison to the MCD and HCD group. We further report a dose-dependent increase in sprout pericyte coverage in ex vivo mouse cultured aortic rings treated with atorvastatin. The direct association between vessel maturity and extravasation of erythrocytes, suggesting that neovessel immaturity leads to IPH is a well know observation [17, 51, 52]. Accordingly, we detected severe areas of IPH in the HCD and MCD groups, but significantly less in HCD + A group. Therefore, our findings indicate that atorvastatin, independently of its lipid lowering effect, reduces intraplaque hemorrhage in ApoE3*Leiden mice vein graft lesion by increasing vessel maturation.

Neovessels within advanced atherosclerotic plaques exhibit compromised EC integrity characterized by the loss of VE-Cad junctions [17]. In our ex vivo cultured mouse aortic rings, VE-Cad expression along the sprouts was largely increased by treatment with atorvastatin. Previous studies have suggested that atorvastatin modulates VE-Cad expression by increasing in VE-PTP transcription [53]. However, others have shown that VE-PTP knockdown does not affect VE-Cad phosphorylation and proteomic analysis did not detect VE-PTP associated with the VE-Cad complex [54]. Moreover, Src kinase has been pointed out as the main implicated mechanism due to its direct association with VE-Cad [55–57]. In our HUVECs cultures, phosphorylation of VE-Cad (Y658) was dose-dependently decreased by atorvastatin, together with its upstream regulator, Src (Y418), when compared with the respective controls. Activation of Src and the subsequent VE-Cad phosphorylation has been described as a critical step in VEGF-induced plasma leakage and its regulated by ANGPT1/2-Tie2 signaling [26, 58, 59]. When ANGPT1 binds to Tie2 receptor, it promotes VE-Cad accumulation on the endothelial cell surface by Src sequestration. However, when ANGPT2 is released, it inhibits Tie2-receptor activation leading to VE-Cad internalization [26]. Interestingly, we also demonstrate that atorvastatin inhibits ANGPT2 release from ECs restoring its Tie2 activation dose-dependently (Fig. 6a–c). However, Tie2 is not exclusively expressed by EC, but also by pericytes [28]. In fact it has been shown that ANGPT2 released from ECs inhibits Tie2 signaling in pericytes, decreasing Akt, activation [28]. Downregulation of the Akt signaling is known to impair pericyte coverage as well as promote hemorrhage and leaky vessel properties. Therefore, such atorvastatin-mediated decrease in ANGPT2 release by EC may explain our in vivo and ex vivo findings on pericyte coverage. Both, intraplaque neovessels from ApoE3*Leiden mice and sprouts from the aortic ring assay, when treated with atorvastatin presented increased pericyte coverage. Thus, we suggest that atorvastatin increases neovessel stabilization by: (1) inhibiting ANGPT2 release from ECs, which (2) restores Tie2-receptor activation in EC and pericytes, and consequently, (3) prevents VE-Cad internalization phospho(Y658)-mediated and pericyte recruitment inhibition.

Taken together, our findings reveal that atorvastatin improves plaque stability by decreasing lesion size and inflammation due to their lipid lowering effect. In addition, atorvastatin also decreases intraplaque angiogenesis and intraplaque hemorrhage independent of changes in the cholesterol levels of ApoE3*Leiden mice. Added to that, atorvastatin increases vessel maturation by improving pericyte coverage and increasing VE-Cad expression. In conclusion, our findings explains the beneficial pleiotropic effects of statins in cardiovascular diseases.
